# Forward and reverse engineering the pain system: from computational neuroscience to neuro-engineering

**DOI:** 10.1097/j.pain.0000000000003705

**Published:** 2025-11

## Abstract

Pain is a complex, multi-level phenomenon integrating sensory, motivational, and cognitive processes. Computational approaches bridge theoretical frameworks with neural and behavioural data, providing descriptive, mechanistic, and normative explanations. We review key computational approaches, including reinforcement learning, control theory, Bayesian inference, and active inference, illustrating their role in understanding pain prediction, avoidance, and modulation. Forward and reverse engineering techniques synergistically refine our models and generate testable hypotheses. This framework not only advances fundamental neuroscience but also informs clinical applications, offering potential for computational phenotyping, personalised therapies, and adaptive neuro-engineering interventions for pain management.

## Why do we need a computational approach to understanding pain?

Theories play an important role in driving neuroscience research of shaping hypothesis generation and constraining the search space for experiments. Computational models bridge theories and observable behaviour, serving as mathematical structures that represent observed data [30]. They sit in direct contrast to a purely data-driven approach, which aims to discover structure in data alone [18]. Thus, computational models offer an intermediary between behavioural theories (e.g., psychology) and physiological accounts (e.g., molecular, cellular, or circuit neuroscience).

Pain can be studied through the lens of its evolutionary and phenomenological components, suggesting a framework based on its adaptive function operating across different spatiotemporal scales. Reaching a common understanding of the data from dramatically different modalities and methodologies requires a multi-level approach, which is afforded by different types of computational models providing different explanations, namely, descriptive, mechanistic and normative explanations. Each of these play distinct roles in building a multi-level account of neural and behavioural phenomena. That is, whenever a new phenomenon is observed, it is often first explained descriptively delineating *what* problem is the brain solving, for example see [7]. This is often followed by mechanistic and normative (or interpretative) explanations delineating *how* is the brain solving it and *why* is the brain solving this problem [9]. We structure the rest of this review around the past, present and future of computational modelling in pain research.

### The advent of computational frameworks for pain

Computational models of pain trace back to early models of learning and conditioning, with roots in classical animal learning theory and its foundational paradigms of Pavlovian and instrumental conditioning [32]. By the late 1990s, theorists developed computational descriptions of these learning phenomena to provide unifying explanations of a variety of behavioural and neural data: most notably the reinforcement learning model of dopamine and reward [40]. Reinforcement learning (RL) is a framework for learning a behavioural policy that maximises cumulative reward (or minimise punishments) over time. This approach was soon extended to explain simple predictive pain learning [43], aligning with fear and aversive conditioning [23], and affective-motivational theories of pain [14,15]. Central to these models was the concept of the prediction error: the difference between what was predicted and what actually occurred, which works as the central teaching signal in these models. RL showed how prediction errors could solve difficult prediction problems [46] across a broad range of learning phenomena involving credit assignment, optimal action learning, and the architecture of pain avoidance learning. Notably, computational parametric human neuroimaging showed that structures such as a striatum and ventromedial prefrontal cortex that had traditionally been thought of as reward-focused, were clearly also involved in pain [44].

In parallel, models of sensory perception, particularly Bayesian perceptual inference offered insights into how prior beliefs shape pain experience, especially in placebo and nocebo effects [6,20,22,26,42,47,51]. These showed how expectations alter pain through perceptual biases, offering a normative account of the sensory-discriminative aspects of pain. However, such perceptual accounts differ from motivational models of endogenous analgesia studied in animals, where pain modulation reflects a flexible, value-based decision that balances competing goals like pain avoidance and reward seeking [13,14]. These motivational models draw from the idea of *value shaping*, in which the perceived value of outcomes is not fixed but flexibly constructed to optimise decisions on-the-fly, paralleling theories in behavioural economics [48]. This approach indeed explained a number of types of endogenous analgesia beyond simple perceptual biases; showing that pain was shaped by both perceptual and motivational factors, which was also implied by the anatomical complexity of descending pathways [3].

### Theory considerations

Here we outline a conceptual map of computational approaches relevant to pain research, offering readers multiple entry points for future modelling efforts. At its core, the brain (and pain system) must solve a difficult control problem under uncertainty — selecting protective actions based on beliefs about the state of the body and world. One influential way to formalise this is by viewing it as a partially observable Markov decision process (POMDP) [25,33], for which Bayesian Decision Theory provides one principled solution [10], and aids as an organising map for understanding the landscape of various computational models. It requires inferring the states or beliefs over states from observations which then is used to learn and direct optimal behaviour with respect to some utilities.

Models focusing primarily on state inference, without control, typically employ Bayesian models of perception [6,47] or special cases of POMDPs such as drift-diffusion models [51]. These reveal how pain perception is shaped by expectations and their modification through learning, and help quantify such effects [51,52]. Recent work extends them to account for biased pain perception (e.g., confirmation bias) [22] and to model sub-optimal learning and information integration in chronic pain [49].

Models emphasising control, while assuming full state observability, simplify the problem to a Markov decision process (MDP) and use reinforcement learning to solve it [46]. Leveraging methods from safe reinforcement learning [17], these models reveal how pain-related control systems guide cautious and self-preserving behaviour [41], inform safe exploration [34] and are being adapted to model maladaptive avoidance [2] and higher punishment sensitivity [35] in chronic pain.

When both inference and control are necessary, full POMDP solutions are required. Some use explicit models of the environment to infer beliefs and plan in belief space (belief MDPs) [31] while others use function approximators like recurrent neural networks to learn belief-like representations through reinforcement learning [19]. These Bayesian decision theoretic models reveal how protective behaviours may obscure evidence of recovery, promoting persistent injury beliefs and transitions to chronic pain—a hypothesis formalised as the *information restriction hypothesis* [33,41].

Early frameworks such as the Helmholtz machine [11] and predictive coding models [16] further provide a recipe for learning and inference in neural networks, informing recent theories of plasticity and back-propagation in the brain [50] and implementation-level accounts of placebo analgesia [6]. Related approaches unified planning and inference, as in Active Inference, where utilities are replaced with preference priors and the agent performs Bayes-optimal behaviour [39]. An algebraic mapping between BDT approaches and active inference approaches is present in control-as-inference [36], KL-control [5] or soft Bellman equations [53].

**Fig. 1:**
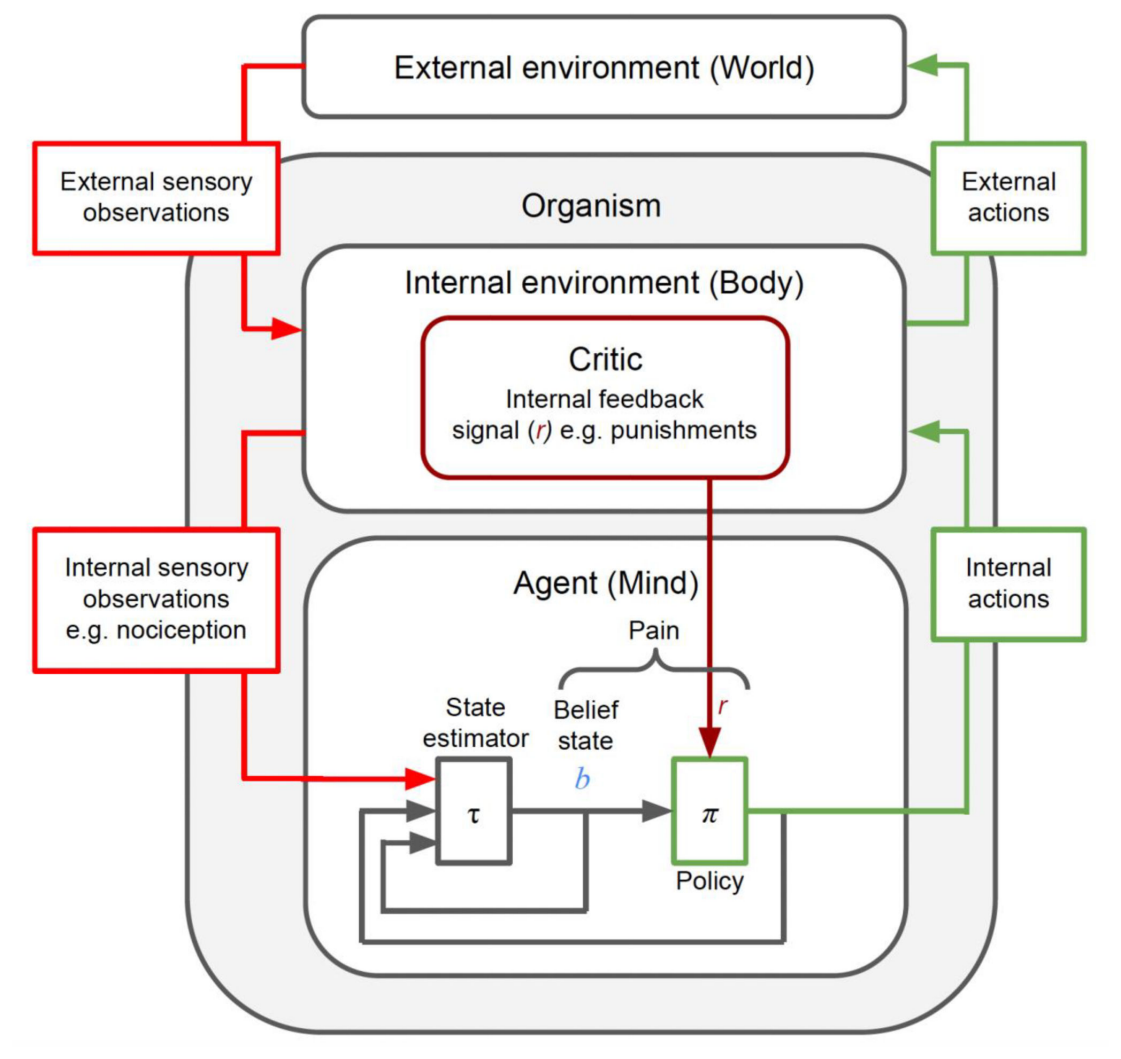
An illustration of computations underlying pain. An internal environment POMDP (e.g. body), which is solved using Bayesian Decision Theory (BDT) by the agent (e.g. mind), necessitating the elements of pain in the mind. The sensory processing of pain is captured via the Bayesian filtering of nociceptive and other relevant sensory observations. The motivational aspect of pain is captured through the precise punishment signal generated and used for temporal credit assignment. Both play a role in affective value learning and subsequent action policy to drive protective behaviour (e.g. pain avoidance).

Framing the pain system’s challenge as a POMDP allows researchers to map different aspects of pain to components such as belief states, value functions, predictive state representations, expected utilities, prediction errors, or Bayesian surprise, facilitating clearer and more testable hypotheses [8,20,21,33,47] (Fig. 1).

### Forward and reverse engineering the pain system

In pain research, *forward engineering* builds models from first principles to simulate behaviour and generate predictions, while *reverse engineering* infers mechanisms by testing specific hypotheses on existing systems (e.g., through experiments). In practice, one can test competing models against behavioural or neural data using techniques like Bayesian model comparison [45]. These findings can then inform forward-engineered models that explain multiple observations across datasets. Such models simulate neural and behavioural responses in diverse paradigms, generating predictions that serve as testable hypotheses. Better data analysis methods and experiments that clearly separate one computation improve reverse engineering attempts, whereas better theories guide what and where to search for and improve forward engineering attempts [24].

As a recent example, we studied how humans combine Pavlovian and instrumental responses to minimise pain/harm [34]. Forward simulations showed that adding Pavlovian fear biases to withdraw enhanced safe learning but at the cost of efficiency in reward acquisition. This led to a normative hypothesis: uncertainty-gated Pavlovian responses could balance safety and efficiency effectively, as demonstrable in simulations. We then tested this prediction in a behavioural task using computational modelling. This illustrates the interplay between the constructivist model building and the reductionist hypothesis testing through carefully designed experiments.

### Clinical applications and neuro-engineering

This approach offers promise for clinical translation. First, forward-engineered models of pain chronification [33] provide principled hypotheses for predicting outcomes and computomics [29] or computational phenotyping [38]. This can support stratification with more robust outcome-prediction biomarkers, with fewer data than brute-force machine learning.

Second, forward-engineered models can be used to design personalised *task controllers* — interactive agents that guide a patient’s learning process in cognitive behavioural therapies (CBT)-like settings [28]. Framed as a two-player game, the controller interacts with a forward-engineered model to best alter the patient’s generative model by guiding the learning process toward desired outcomes such as improved motivation, reduced avoidance, or resilience to stress. This builds on ideas from curriculum learning [4] and shaping [27].

Third, biases in pain and injury perception may affect post-injury behaviour [49] which could contribute to phenomena like boom-bust cycles in chronic pain [1,37]. Personalised models of temporal patterns in activity and pain perception could enhance activity pacing interventions by offering more targeted routine guidance.

Fourth, information-rich models beget information-rich solutions, such as functionally targeted neurotechnologies [12]. This dovetails with an understanding of how generative processes lead to biomarkers, allowing closed loop technologies that tune the intervention to the outcome. Ultimately, systems engineering approaches can integrate multiple sensing and interventional technologies (alongside drugs), utilising cognitive and behavioural platforms, yielding holistic therapies that exploit the combined strength of multiple individual approaches.
